# Longitudinal Serum Metabolomics in Extremely Premature Infants: Relationships With Gestational Age, Nutrition, and Morbidities

**DOI:** 10.3389/fnins.2022.830884

**Published:** 2022-02-17

**Authors:** Anders K. Nilsson, Abdellah Tebani, Daniel Malmodin, Anders Pedersen, Gunnel Hellgren, Chatarina Löfqvist, Ingrid Hansen-Pupp, Mathias Uhlén, Ann Hellström

**Affiliations:** ^1^Section for Ophthalmology, Department of Clinical Neuroscience, Institute of Neuroscience and Physiology, Sahlgrenska Academy, University of Gothenburg, Gothenburg, Sweden; ^2^Department of Metabolic Biochemistry, UNIROUEN, INSERM U1245, CHU Rouen, Rouen University Hospital, Normandie University, Rouen, France; ^3^Department of Protein Science, Science for Life Laboratory, KTH-Royal Institute of Technology, Stockholm, Sweden; ^4^Swedish NMR Centre, University of Gothenburg, Gothenburg, Sweden; ^5^Institute of Biomedicine, Sahlgrenska Academy, University of Gothenburg, Gothenburg, Sweden; ^6^Institute of Health and Care Sciences, Sahlgrenska Academy, University of Gothenburg, Gothenburg, Sweden; ^7^Department of Clinical Sciences, Pediatrics, Skåne University Hospital, Lund University, Lund, Sweden

**Keywords:** bronchopulmonary dysplasia, enteral nutrition, parenteral nutrition, retinopathy of prematurity, ketone bodies, ethanolamine, one-carbon metabolism, human milk

## Abstract

An increasing number of extremely premature infants survive the neonatal period and beyond. Little is known about the maturation of the preterm infant’s metabolome and its relation to the development of morbidities. Using 1H-NMR, we investigated the serum metabolic profile of 87 infants born at a gestational age (GA) <28 weeks [mean GA (SD) 25.4 (1.4) weeks] in samples longitudinally collected from birth to term equivalent age. The infant metabolome was analyzed in relation to GA, postnatal age, nutrition, and preterm morbidities. At postnatal day 1, low GA correlated with high levels of 3-hydroxyisobutyrate, acetate, acetoacetate, acetone, formate, glucose, and valine. Nearly all quantified metabolites displayed postnatal concentration changes. For example, the two phospholipid-related metabolites myo-inositol and ethanolamine displayed a similar decline from birth over the first weeks of life, irrespectively of GA. The proportion of enteral/parenteral energy intake in the first 28 days significantly correlated with mean levels of 52% of the analyzed metabolites. Low enteral energy intake was associated with high serum levels of 3-hydroxyisobutyrate, creatinine, glucose, glycerol, histidine, lactate, leucine, lysine, methionine, ornithine, phenylalanine, proline, threonine, and uridine. There were also significant correlations between high enteral intake and high serum levels of isoleucine and tyrosine. Retinopathy of prematurity (ROP) and bronchopulmonary dysplasia (BPD) outcomes were not significantly associated with metabolite levels in the neonatal period after correcting for multiple testing. In conclusion, the serum metabolome of extremely premature infants changes substantially in the neonatal period, largely driven by the gradual transfer from total parenteral nutrition to full enteral feeding. Further studies are needed to disentangle the intricate relationships between the metabolome, nutritional management, GA, and the development of preterm morbidities.

## Introduction

Advances in obstetrics and perinatal care over the last decades have dramatically improved the survival of infants born preterm. Despite improved survival, the prevalence of morbidities among extremely preterm infants (born <28 weeks of gestation) remains high, particularly among infants born at the lowest gestational ages (GAs) (Costeloe et al., 2012; Norman et al., 2019). Morbidities affecting preterm infants include intraventricular hemorrhage (IVH), necrotizing enterocolitis (NEC), sepsis, bronchopulmonary dysplasia (BPD), and retinopathy of prematurity (ROP). These morbidities pose immediate threats to infant health and may have long-term consequences for neurodevelopmental and behavioral outcomes (Mathewson et al., 2017; Pascal et al., 2018; Twilhaar et al., 2018). The neonatal period is an especially vulnerable time for extremely preterm infants with high risks for death and morbidities.

Metabolomics allows for the detection and quantification of an array of low molecular weight compounds derived from cellular metabolism or exogenous sources and has the potential to provide detailed insights into disease etiology and progression (La Frano et al., 2018; Ernst et al., 2020). Metabolomics is increasingly used in research settings and is a promising tool in precision medicine, especially in the context of clinical neonatology (Fanos et al., 2018). However, most metabolomics studies in preterm infant blood have focused on one or a few sampling occasions, while longitudinal studies during the neonatal period and beyond have been sparse and included few individuals.

The neonatal period of extremely preterm infants involves the gradual transfer from total or partial parenteral nutrition (PN) to full enteral nutrition (EN). Human milk is always the firsthand choice of enteral feed for preterm infants. In Sweden, all extremely preterm infants receive exclusively human milk, mother’s own milk if available, otherwise, donor human milk, from the day of birth until usually 34 weeks postmenstrual age. Thereafter, a preterm formula may be introduced. Feeding tolerance is strongly linked to the GA at birth, and the most immature infants require the longest time to reach full enteral feeding. Therefore, an infant’s metabolic profile depends on the GA and the postnatal age (Clark et al., 2014). Furthermore, the feeding regime influences the growth trajectory for the preterm infant (Power et al., 2019; Lund et al., 2020; Roggero et al., 2020), and poor growth gain during the 1st weeks of life is predictive for adverse morbidity outcomes, including severe ROP and BPD (Hellström et al., 2010; Chisholm et al., 2019).

Bronchopulmonary dysplasia and ROP are multifactorial diseases with certain common elements but also with distinct pathogenesis. ROP is a neurovascular disease characterized by dysregulated retinal vascularization that can lead to retinal detachment and permanent visual loss if not timely diagnosed and treated. The prevalence of ROP is higher in infants with low GA and low weight at birth (Hellström et al., 2013; Danielsson et al., 2021). BPD stems from pre and postnatal injury to the immature lung and affects almost 50% of extremely preterm infants born in high-income countries (Stoll et al., 2010; Hellström et al., 2021; Siffel et al., 2021). As for ROP, BPD involves disrupted vascular formation and is more common in infants with low GA and high supplemental oxygen.

Here, we assessed the serum metabolic profiles of extremely preterm infants from birth to term equivalent age and analyzed the metabolome in regards to GA, postnatal age, nutrition, and morbidities.

## Materials and Methods

### Study Design

The current investigation was performed within the Donna Mega trial (ClinicalTrials.gov Identifier: NCT02760472). The Donna Mega trial is a single-center, randomized open-label trial designed to determine the effect of parenterally administered lipids with or without *n*–3 long-chain polyunsaturated fatty acids (LC-PUFAs) on growth and morbidity outcomes in infants born extremely preterm. Infants were randomly assigned an olive oil-based lipid parenteral emulsion (Clinoleic, Baxter Medical AB, Kista, Sweden) or a mixed-oil emulsion high in *n*–3 LC-PUFAs (Smoflipid, Fresenius Kabi, Uppsala, Sweden). The results of the primary outcomes of the trial have been previously published (Najm et al., 2017). Eligible for inclusion in the study were infants born at GA <28 weeks at the neonatal care unit at Sahlgrenska University Hospital in Gothenburg, Sweden, between April 2013 and September 2015. Infants born with major congenital malformations were excluded. Out of the 138 infants born at GA <28 weeks during the inclusion period, parents/legal guardians of 90 eligible infants agreed to participate after informed consent. Details of the nutritional strategy and nutritional data collection have been described in detail (Najm et al., 2017; Lund et al., 2020). Growth data (weight, length, and head circumference) were prospectively collected until term equivalent age and transformed into z-scores according to the growth reference by Niklasson and Albertsson-Wikland (2008).

### Morbidities

Retinopathy of prematurity screening was performed as previously described (Löfqvist et al., 2018), and disease stages were classified using The International Classification of Retinopathy of Prematurity (2005). No/moderate ROP was defined as ROP stages 1–2, and Severe ROP as ROP stage 3 and/or Type 1 ROP. BPD was defined as the need for supplemental oxygen at 36 weeks postmenstrual age.

### Blood Collection, Sample Preparation, and Metabolic Profiling

Infant blood samples were collected at postnatal days (PND) 1, 7, 14, and 28, and at postmenstrual age (PMA) 32, 36, and 40 weeks. The blood was allowed to clot at 4°C for a minimum of 45 min and a maximum of 2 h before centrifugation at room temperature at 1500 *g* for 10 min. The serum was then transferred into cryovials and stored at −20°C for up to 1 week before long-term storage at −80°C until analysis. All samples had been subjected to at least one, but less than five, freeze-thaw cycle before NMR analysis.

Serum samples (50 μl) were prepared and then analyzed on an Oxford 800 MHz magnet equipped with an Avance III HD console and 3 mm TCI cryoprobe (Bruker BioSpin). Details of sample processing and the ^1^H NMR analysis have been published (Nilsson et al., 2020). Tentative annotations of peaks were made using ChenomX 8.3 (ChenomX Inc.) and the spectral data in the Human Metabolome Data Bank (Wishart et al., 2018). In total, 260 NMR features were detected in the serum samples. From these, 31 metabolites could be chemically and structurally annotated. In instances where multiple features represented a single metabolite, a strong well-separated peak was selected, or two or more peaks were summarized. All metabolite levels are reported as normalized NMR signals or as standardized values.

### Ethics

The Donna Mega trial was approved by the Regional Ethical Review Board, Gothenburg (Dnr 303-11). Informed signed consent for all participants included in the study was obtained from their parents or legal guardians.

### Statistical Analyses

Mann–Whitney *U*-test was used to compare continuous variables with non-normal distribution, and Spearman’s rank test was applied to assess correlations between variables.

The area under the curve (AUC) for metabolites for PND 1–28 was calculated using the trapezoidal rule. The AUC was divided by the total number of days to yield a daily mean value. Only infants who had a complete sample series during their first 4 weeks of life (PNA 1, 7, 14, and 28) were included in this sub-analysis. In instances where no NMR signal could be detected and a peak intergraded, the minimum value/2 was imputed. Six out of 15593 (0.04%) values were imputed using this method.

For clustering, the NMR signal of each metabolite was first standardized with a standard deviation of 1 centered at 0. Then, the scaled values from all 503 samples were used to create the Euclidean distance matrix for dendrogram generation. Dendrograms showing metabolite levels in heatmaps have been clustered using the Ward2 algorithm, an implementation of Ward’s minimum variance method implemented as “Ward.D2” in R package pheatmap (Kaufman and Rousseeuw, 2009; Murtagh and Legendre, 2014). Spearman correlation was used to compute the pairwise correlation. Multiple tests correction was performed with the Benjamini–Hochberg method.

Statistical analyses were performed using IBM SPSS Statistics version 27 (IBM Corp., Armonk, NY, United States) and R-Statistics Software (The R Foundation for Statistical Computing, Vienna, Austria).

## Results

### Population Characteristics and Serum Sampling Scheme

**FIGURE 1:**
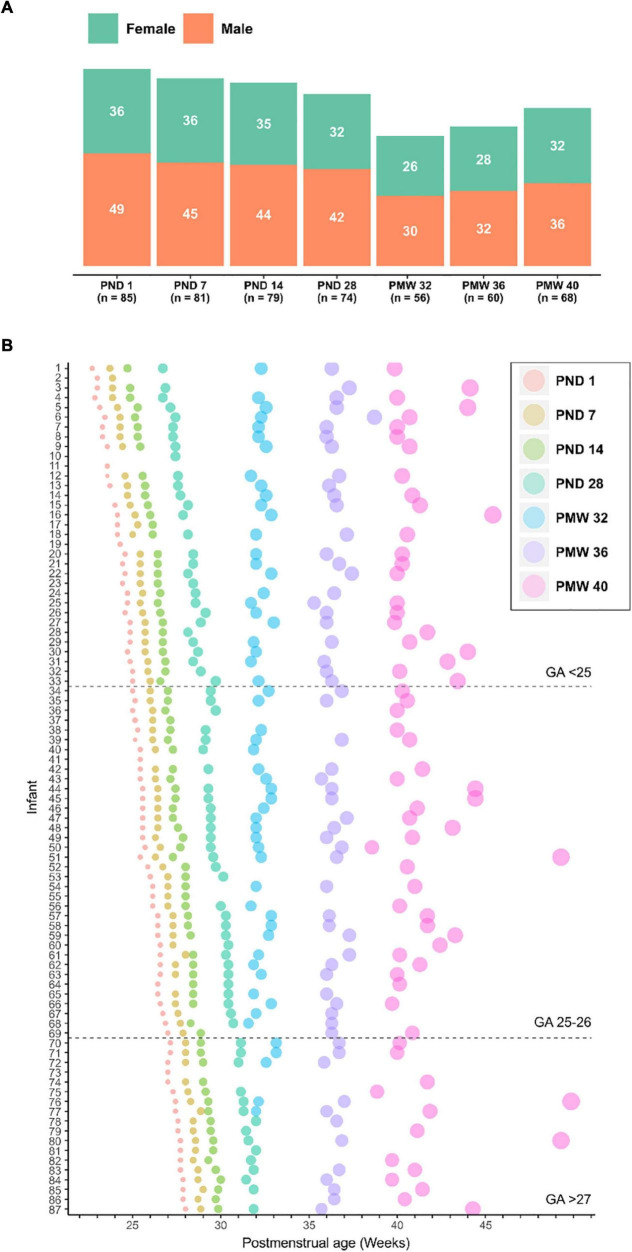
Overview of analyzed samples. **(A)** Number of samples collected at seven time points used for serum metabolomics presented by gender. **(B)** Longitudinal serum sampling scheme for all infants included in the study. Infants are sorted by gestational age at birth. The size of the dot at sampling is proportional to the postnatal age of the infant and the color indicates the sampling time point accordingly to legend. GA, gestational age; PND, postnatal day; PMW, postmenstrual week.

Serum for ^1^H NMR metabolomics was available from 87 out of 90 infants enrolled in the Donna Mega trial (Najm et al., 2017). The study infants’ demographic and clinical data are presented in Table 1 and the blood sampling scheme used is illustrated in Figure 1. In total, 503 serum samples collected at seven predetermined time points were analyzed (Figure 1A). Samples from the first four time points were collected based on chronological age [postnatal day (PND) 1, 7, 14, and 28], while the following samples were collected based on postmenstrual age [postmenstrual week (PMW) 32, 36, and 40) (Figure 1B).

**TABLE 1:** Demographic data of the study population (*n* = 87).

Variable	Value
Gestational age at birth, mean (SD), wks	25.4 (1.4)
Birth weight, mean (SD), g	780 (224)
Birth zWeight, mean (SD)^a^	−0.86 (1.36)
Birth weight small for gestational age^b^, *n* (%)	12 (14)
Male, *n* (%)	50 (57)
Retinopathy of prematurity (ROP), stages 0–2	47/78
Retinopathy of prematurity (ROP), stages 3 or Type I	31/78
Bronchopulmonary dysplasia (BPD)^c^, *n* (%)	39/78 (50)
Death before 40 weeks PMA, n (%)	9 (10)

*^a^Calculated according to Niklasson and Albertsson-Wikland (2008).*

*^b^BW <−2 SD.*

*^c^Supplemental O_2_ at 36 weeks PMA.*

### Overview of the Infant Metabolome

**FIGURE 2:**
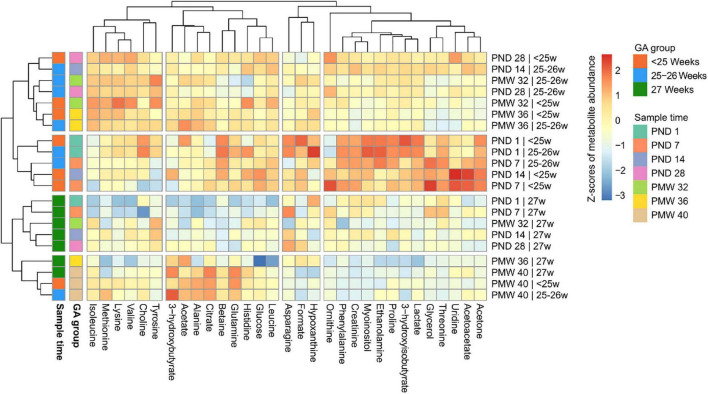
Heatmap of metabolite levels over the study period stratified by gestational age (GA) at birth: <25 weeks, 25–26 weeks, or 27 weeks. Each metabolite was scaled using the mean value and standard deviation and centered at 0. Intensities from blue to red display relative metabolite levels in standard deviations. Both rows and columns have been hierarchically clustered based on metabolite abundances at specified time points for each GA group. Thus, metabolites that show similar abundance patterns across the study period group together vertically, and time points for each GA group that show similar metabolite abundance patterns group horizontally. Breaks in the heatmap indicate clusters from the dendrograms; the number of clusters was manually specified. PND, postnatal day; PMW, postmenstrual week.

Thirty-one serum metabolites could be unambiguously annotated and quantified in all samples (Supplementary Table 1). To overview the data, mean metabolite levels across the sample time points were subjected to hierarchical clustering and visualized in a heatmap (Figure 2). For assessing the impact of GA on the metabolome, infants were subdivided into three groups based on GA at birth: below 25 weeks GA (*n* = 33), 25–26 weeks GA (*n* = 36), and 27 weeks GA (*n* = 18). There was apparent clustering both according to sampling time and GA at birth. Overall, samples from the two lower GA groups (<25 and 25–26 weeks) clustered together and showed larger variability in metabolite levels between time points than samples from the group of infants with the highest GA (27 weeks). Samples collected at PND 1 and PND7 in the two lower GA groups formed a cluster defined by high levels of acetone, creatinine, and phenylalanine. Furthermore, PND 1 samples from GA <27 weeks infants displayed high 3-hydroxyisobutyrate, ethanolamine, and myoinositol levels, among other metabolites. At PMW 40, samples from all three GA groups clustered together, indicating a transient change in the metabolome in infants born at the lowest GAs occurring in the neonatal period.

### Influence of Gestational Age at Birth on Metabolites the First Day of Life

**FIGURE 3:**
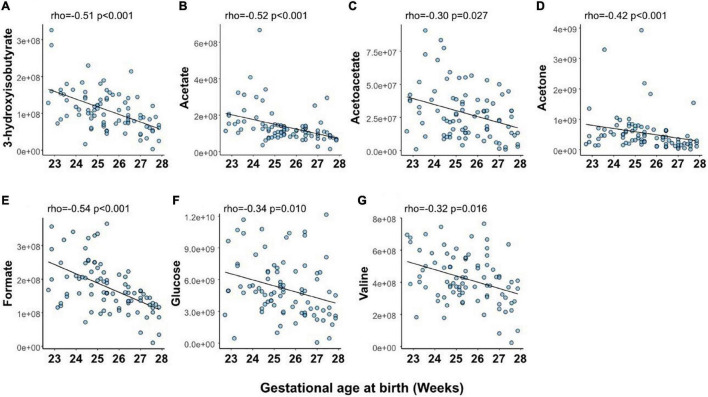
Relationship between gestational age at birth and serum levels of metabolites at postnatal day (PND) 1: 3-hydroxyisobutyrate **(A)**, acetate **(B)**, acetoacetate **(C)**, acetone **(D)**, formate **(E)**, glucose **(F)**, and valine **(G)**. Shown are Spearman’s correlation coefficient (rho) and *p*-value for the association between metabolite levels and gestational age. *p*-values have been false discovery rate adjusted using the Benjamini-Hochberg method. *Y*-axis shows normalized NMR signal.

At PND 1, levels of 7 (23%) of the analyzed metabolites were significantly correlated with GA at birth (Figure 3 and Supplementary Table 2). Notably, all metabolites associated with GA at birth displayed negative correlations.

### Longitudinal Changes in the Infant Metabolome

**FIGURE 4:**
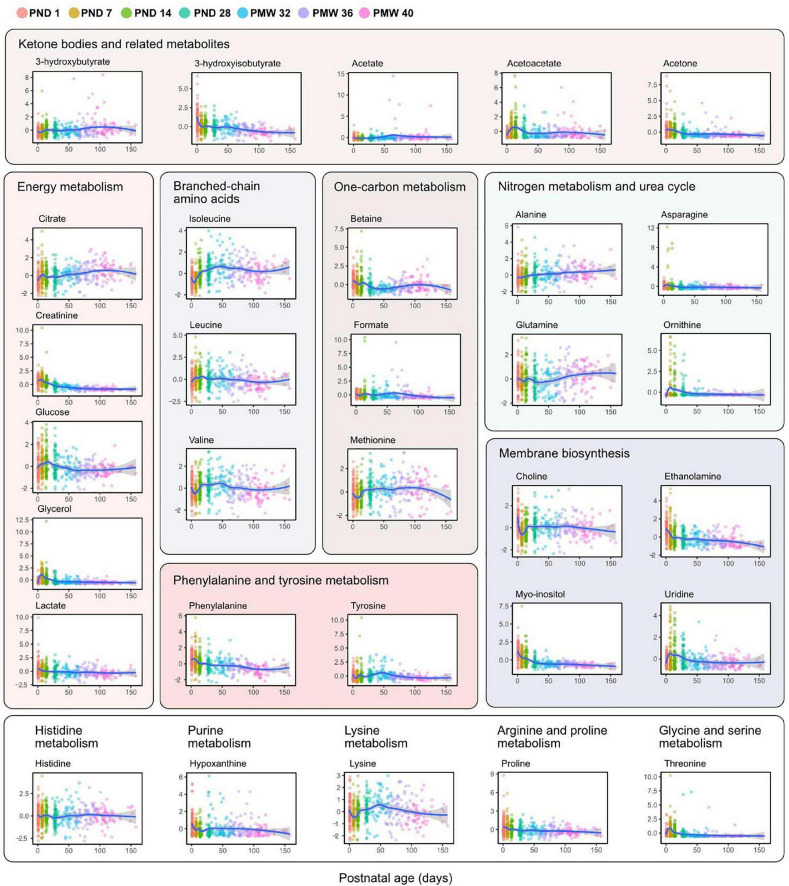
Metabolite levels over postnatal age (days). Individual sample values are plotted and colored according to time point as indicated in the legend. Lines represent smoothed conditional means with 95% confidence intervals (shaded areas). *Y*-axis shows z-score normalized metabolite abundances.

**FIGURE 5:**
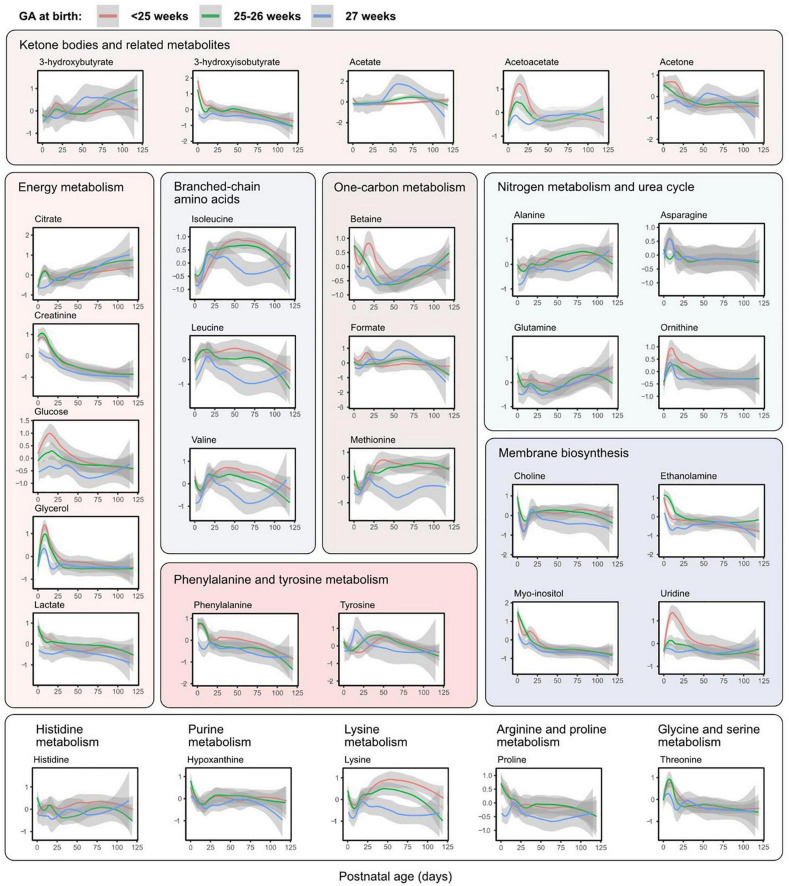
Metabolite levels over postnatal age (days) by gestational age (GA) group. Accordingly to the legend, metabolite levels were stratified by GA at birth. Lines represent smoothed conditional means with 95% confidence intervals (shaded areas). Means are only shown up to day 120 due to few samples in each group at later time points. Y-axis shows z-score normalized metabolite abundances.

Figure 4 shows all analyzed metabolites longitudinally plotted with individual samples colored according to time point. The same metabolites presented by the three GA groups are shown in Figure 5. As seen in both the heatmap (Figure 2) and longitudinal plotting of the data (Figures 4, 5), the most pronounced metabolome changes occurred during the neonatal period, i.e., between birth and postnatal day 28. Specifically, this period was also defined by differences in metabolite levels between the three GA groups. Some metabolites, however, also displayed abundance changes in the later part of the study period (e.g., citrate, glutamine, and lysine).

### Relationships Between Nutritional Management, Growth, and the Infant Metabolome

**FIGURE 6:**
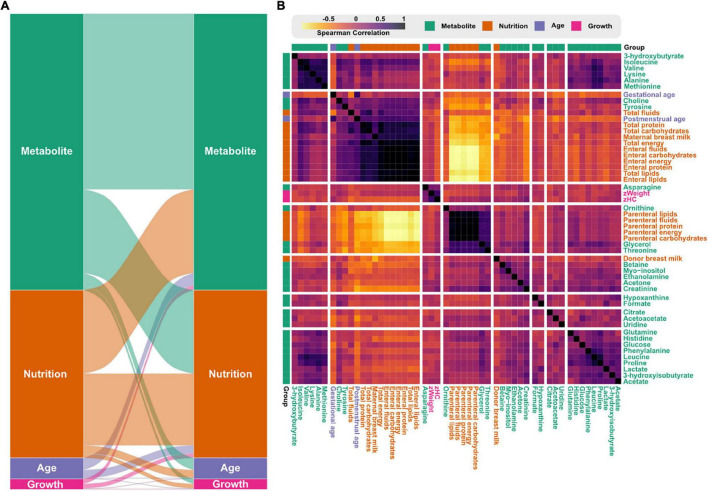
Correlations between nutritional intake, growth, and the infant serum metabolome. **(A)** Flow diagram showing the correlations within and between variables groups based on Spearman correlations above 0.5. Link-width is proportional to the number of features correlating between the variable groups. **(B)** Heatmap with hierarchical clustering based on the pairwise Spearman correlations between variables. Color reflects the Spearman’s rank correlation coefficient accordingly to the legend.

Next, we explored relationships between the infant metabolome, growth, and nutritional parameters by correlating data collected the first 28 postnatal days (Figure 6). About half of the nutritional variables were significantly associated with metabolite levels (Figure 6A). Variables related to parenteral nutrition formed a cluster and were positively correlated with serum levels of glycerol, threonine, and ornithine (Figure 6B). Contrary, nutritional variables related to enteral nutrition formed a cluster together with choline and tyrosine. Surprisingly, donor milk and mother’s own milk were found in two separate clusters. Standardize weight (zWeight) and head circumference (zHC) formed a small cluster together with asparagine.

### Enteral Nutritional Intake and the Serum Metabolome

**FIGURE 7:**
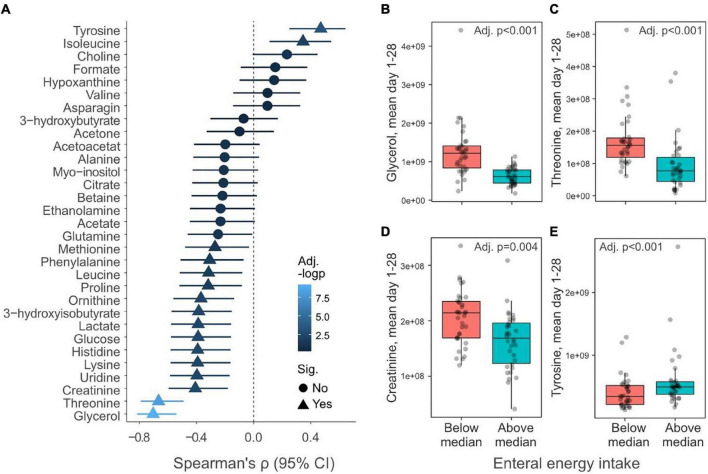
Relationship between enteral nutrition and serum metabolites. **(A)** Spearman correlation (95% confidence interval) between energy provided as enteral nutrition day 1–28 and mean daily metabolite levels during the corresponding period (*n* = 69). Triangles indicate metabolites that significantly (*p* < 0.05) correlate with enteral energy intake and the color of the symbols reflect the negative logarithm of the *p*-value. **(B–E)** Boxplots show mean daily levels of selected metabolites that depend on the total energy provided as enteral energy. Infants have been dichotomously grouped if they received above (*n* = 34) or below (*n* = 35) the median amount of total energy as enteral energy day 1–28. *Y*-axis shows normalized NMR signal. *p*-values have been false discovery rate adjusted using the Benjamini–Hochberg method.

To further understand how nutritional management influences the metabolome, we compared the % enteral of total energy intake (kcal/kg/d) PND 1–28 with mean metabolite levels. Only infants with complete metabolic profiles from PND 1 to PND 28 were included (*n* = 69). Isoleucine and tyrosine displayed positive correlations with % enteral energy intake, whereas many other (14/31) metabolites were negatively associated, including glycerol, threonine, and creatinine (Figure 7A). Next, infants were dichotomously grouped based on if they had high (>median) or low (<median) enteral energy intake PND 1–28 (Supplementary Table 3). Figures 7B–E show examples of metabolites that significantly differed between infants with high and low enteral energy intake. Among these examples, the daily mean glycerol and threonine levels were two-fold higher in infants who received little enteral intake compared to infants with high enteral intake.

### Associations Between the Serum Metabolome and Morbidities

To identify metabolites associated with the development of preterm morbidities, we compared mean daily metabolite levels PND 1–28 with ROP and BPD outcomes. Before correction for multiple testing, 10 metabolites differed significantly between infants who developed no/mild ROP (stages 0–2, *n* = 39) and infants who developed severe ROP (stage 3 and/or Type 1 ROP, *n* = 29) (Supplementary Table 4). However, after false discovery rate correction, no metabolite remained statistically significant. Similarly, three metabolites differed significantly between infants who did not (*n* = 36) or did develop BPD (*n* = 32), but none remained significant after false discovery rate correction (Supplementary Table 4).

## Discussion

We longitudinally followed the serum metabolome of extremely preterm infants from birth to term equivalent age. Our results show that the metabolome of the infants undergoes substantial composition changes during the early perinatal period, strongly influenced by the feeding regime, and moreover, relates to GA at birth.

The levels of seven serum metabolites on PND 1 significantly correlated with GA at birth (3-hydroxyisobutyrate, acetate, acetoacetate, acetone, formate, glucose, and valine). Valine has previously been shown to be the amino acid with the most considerable concentration difference between extremely preterm and full-term infants, demonstrating a GA dependence (Wilson et al., 2014). Apart from valine, the identified metabolites constituted non-amino acid compounds and showed limited overlap with metabolites related to GA in very- and moderately preterm infants (Ernst et al., 2020). Formate can be produced from various sources and is a critical component in one-carbon metabolism, including the metabolic pathways of folate, choline, and methionine. The concentration of formate is higher in fetal plasma than in maternal plasma (Washburn et al., 2015; Brosnan et al., 2019). It has been suggested that high fetal formate supports increased one-carbon metabolism during late gestation when extensive growth occurs (Brosnan et al., 2019). We found that low GA at birth was associated with higher serum formate at PND 1. The implications of this finding need further studies. Acetoacetate, acetone, and 3-hydroxybutyrate are ketone bodies that generally increase in the blood under fasting conditions, a high-fat diet, suckling, and during the second half of the pregnancy. Ketone bodies are formed from the breakdown of fatty acids and are used as fuel when glucose is unavailable or needed for anabolism. Further metabolism of acetone yields lactate and acetate, among other compounds. Ketone bodies can freely cross the placenta, and maternal-fetal concentrations are closely related at delivery (Paterson et al., 1967). We found infant ketone levels at PND 1 to be lower in infants born at higher GAs. The increase in acetoacetate observed at PND 7 and 14, especially in lower GAs, likely reflects a high intake of parenteral lipids and increased plasma lipid levels.

Serum myo-inositol and ethanolamine followed a similar decline after birth to reach plateau levels around PND 28. Both of these metabolites are essential components of membrane phospholipids. A postnatal decrease in myo-inositol is known to occur in preterm infants (Brion et al., 2021), and attempts have been made to restore myo-inositol levels to reduce morbidity, particularly respiratory distress syndrome and ROP (Hallman et al., 1992; Phelps et al., 2018). However, a recent systematic review concluded that myo-inositol supplementation to preterm infants did not reduce adverse outcomes (Howlett et al., 2019). To the best of our knowledge, decreasing serum levels of ethanolamine following extremely preterm birth has not previously been reported, although urine metabolomics has identified ethanolamine to decline with postnatal age in full-term infants (Scalabre et al., 2017). Ethanolamine is a precursor for the synthesis of phosphatidylethanolamine and phosphatidylcholine, the two most abundant phospholipids in humans, and circulating ethanolamine can act as a growth factor to stimulate hepatocyte proliferation (Sasaki et al., 1997; Kume et al., 2006). The consequences of a postnatal loss of serum ethanolamine in preterm infants warrant further research.

We correlated infant serum metabolites with growth parameters and nutritional data collected over the first 4 weeks of infant life. Choline, a compound involved in one-carbon metabolism and phospholipid synthesis, correlated positively with enteral nutrition. Choline is not present in PN solutions, and we previously reported that high parenteral intake is associated with lower infant serum choline (Nilsson et al., 2020). Somewhat surprisingly, the mean intake (ml/kg) of human donor and mother’s own milk, respectively, were grouped into two separate clusters. This might reflect differences in the nutritional composition between mother’s own milk and donor milk [reviewed in Hård et al. (2019)], or reflect that the two types of milk are used to different extents during the neonatal period. The strongest (positive) association for z-score weight and head circumference was with asparagine. Other studies have identified metabolites, included asparagine, to be associated with poor extrauterine growth in preterm infants (Dudzik et al., 2020; Wang et al., 2020). Both of these studies found higher asparagine levels in well-grown infants compared to infants with extrauterine growth restriction, which matches our data.

Detailed analysis of the relationship between nutrition and metabolites revealed that a majority of the analyzed amino acids in serum were negatively correlated with enteral energy intake (i.e., positively related to parenteral nutrition intake). This finding is expected and in line with previous reports showing an increase in infant blood amino acids after parenteral administration of amino acids (Clark et al., 2007; Blanco et al., 2011). Furthermore, glucose and glycerol levels were similarly related to parenteral nutrition intake, likely reflecting glucose and parenteral lipid administration.

Previous efforts have been made to detect disturbances in metabolic pathways relating to ROP and BPD development. However, the results of these studies have not been conclusive; for studies on ROP, differences likely relate to clinical characteristics of the subjects, timing of sample collection, and analysis platform (Yang et al., 2020; Zhou et al., 2020). In an animal model for ROP, oxygen-induced retinopathy, arginine and proline pathways were identified to be dysregulated and proposed as biomarkers for human ROP (Paris et al., 2016; Lu et al., 2020). Blood, urine, and tracheal aspirate have been used as matrixes in search of metabolites relating to BPD (La Frano et al., 2018; Lal et al., 2018; Pintus et al., 2018; Piersigilli et al., 2019). As for ROP, the results from these studies have been inconclusive and several unrelated groups of metabolites have been pointed out as indicative for BPD development. We compared mean daily metabolite levels in the first 4 weeks of life with later development of severe ROP or BPD. While our analysis failed to identify metabolites related to ROP or BPD that could withstand false discovery rate correction, 10 of the analyzed metabolites were significantly associated with ROP outcome and three metabolites with BPD outcome before multiple testing corrections. We believe that these metabolites merit further discussion for hypothesis generation, keeping in mind the apparent risk of statistical Type 1 errors. There was considerable overlap between metabolites related to ROP outcome and enteral energy intake; metabolites associated with severe ROP tended to be negatively associated with enteral energy intake (3-hydroxyisobutyrate, glucose, glycerol, lactate, lysine, myoinositol, phenylalanine, and uridine). Moreover, several metabolites related to GA at PND 1 were also associated with severe ROP outcome (3-hydroxyisobutyrate, acetoacetate, glucose, lactate, and phenylalanine). We interpret this as a reflection of high parenteral nutrition intake as well as low GA in infants who develop severe ROP. Low energy intake *per se* in the 1st weeks of life is a known risk factor for ROP (Stoltz Sjöström et al., 2016; Klevebro et al., 2019). Metabolites associated with BPD severity (before adjusting for multiple testing) overlapped with those associated with ROP severity and included glucose, lactate, and uridine. In line with these results, severe BPD and severe ROP have been reported to be significantly related after adjustment for GA and other confounders (Podraza et al., 2018; Singh et al., 2019), and the two diseases share several pathogenic factors (Stark et al., 2018). As no metabolite was found to be significantly associated with ROP or BPD after correcting for multiple testing, we did not proceed to adjust for GA, nutritional intake, or other known confounders in our analyses. GA and parenteral nutrition are intimately linked: the transition to full enteral feeding proceeds slower in infants born at low GA. Thus, it is challenging to disentangle metabolomic alterations related to preterm disease and those that reflect GA-related nutritional management.

There are limitations to our study. Serum samples used in this study were subjected to one or a few freeze-thaw cycles before analysis, which is a known factor to impact the metabolome (Pinto et al., 2014). However, intra-individual differences in metabolic profiles have been reported to be smaller than inter-individual differences, i.e., the variability between infants is more extensive than can be expected due to sample handling (Hirayama et al., 2015). Further, although we used an untargeted metabolomics approach, NMR analysis primarily identifies compounds in the micromolar range, introducing a bias to the type of metabolites that are quantified. Finally, this study included a limited number of infants, and larger prospective studies are needed to elucidate the intricate relationships between the metabolome, environmental factors, and prematurity-related disorders. One of the strengths of this study lies in its longitudinal design. Few, if any, studies have previously followed the metabolome of preterm infants over the neonatal period with comparable high temporal resolution.

## Conclusion

We provide a comprehensive view of the evolution of the serum metabolome of extremely preterm infants from birth to term equivalent age. There was large interindividual variability in the metabolome that was partly explained by the GA at birth and by the amount of enteral and parenteral nutrition the infant received. The results from our and other studies imply that there is not a single metabolic biomarker that could satisfactorily distinguish those infants who are at the highest risk to develop ROP or BPD. Instead, enhanced diagnosis of ROP and BPD must likely rely on integrative and multiparameter biosignatures that encompass the complexity of these diseases.

## Data Availability Statement

The original contributions presented in the study are included in the article/Supplementary Material, further inquiries can be directed to the corresponding author.

## Ethics Statement

The studies involving human participants were reviewed and approved by The Regional Ethical Review Board, Gothenburg. Written informed consent to participate in this study was provided by the participants’ parents or legal guardians.

## Author Contributions

AN, GH, and AH contributed to the conception of the research. AP and DM contributed to the data acquisition. AN, AT, and DM contributed to the analysis of the data. AN drafted the manuscript. All authors critically revised the manuscript, agreed to be fully accountable for ensuring the integrity and accuracy of the work, read and approved the final manuscript, contributed to the design of the research, and contributed to the interpretation of the data.

## Conflict of Interest

The authors declare that the research was conducted in the absence of any commercial or financial relationships that could be construed as a potential conflict of interest.

## Publisher’s Note

All claims expressed in this article are solely those of the authors and do not necessarily represent those of their affiliated organizations, or those of the publisher, the editors and the reviewers. Any product that may be evaluated in this article, or claim that may be made by its manufacturer, is not guaranteed or endorsed by the publisher.
